# Semaglutide and Non-arteritic Anterior Ischemic Optic Neuropathy: A Systematic Review

**DOI:** 10.7759/cureus.89656

**Published:** 2025-08-08

**Authors:** Roberto A Hidalgo Ramos, Marcelo Ortiz, Sebastián Dufner Krieger, Daniela Secades

**Affiliations:** 1 Faculty of Medicine, University of Costa Rica, San Jose, CRI

**Keywords:** diabetes, glp-1 receptor agonist, naion, optic neuropathy, semaglutide

## Abstract

This systematic review examines the potential association between semaglutide, a glucagon-like peptide-1 (GLP-1) receptor agonist, and the development of non-arteritic anterior ischemic optic neuropathy (NAION). Nine studies were included, consisting of retrospective cohort analyses, case series, and pharmacovigilance reports. Findings across the literature were inconsistent, with some studies reporting an increased risk while others found no significant association. Variations in study design, diagnostic criteria, and population characteristics limited comparability and prevented meta-analysis. Overall, the absolute risk appears to be low, and the therapeutic benefits of semaglutide in managing type 2 diabetes and obesity are likely to outweigh potential ocular concerns for most patients. Clinicians should exercise caution in individuals with predisposing ocular risk factors, such as small Bruch’s membrane opening (BMO), optic disc drusen (ODD), or otherwise crowded optic nerve heads. Further prospective studies with rigorous ophthalmologic evaluation are needed to clarify any causal relationship.

## Introduction and background

Glucagon-like peptide-1 receptor agonists (GLP-1 RAs) represent a significant class of medications primarily used in the management of type 2 diabetes mellitus (T2DM) and, increasingly, obesity [1-3]. These agents, including semaglutide, liraglutide, dulaglutide, exenatide, and lixisenatide, are synthetic analogs of the natural human hormone GLP-1, with enhanced pharmacokinetic properties that allow for more stable and prolonged effects [1-3]. They work by enhancing glucose-dependent insulin release, inhibiting glucagon output, delaying gastric emptying, and promoting satiety [2-3]. These combined actions result in improved glycemic control with minimal risk of hypoglycemia and clinically meaningful weight loss [2].

In addition to metabolic effects, GLP-1 RAs are cardioprotective and renoprotective, lowering the risk of major cardiovascular events such as stroke and improving renal health, although the full mechanisms remain unclear [2-3].

Despite these therapeutic benefits, recent reports have suggested a possible link between GLP-1 RAs, especially semaglutide, and non-arteritic anterior ischemic optic neuropathy (NAION) [1]. NAION is the most common type of ischemic optic neuropathy (ION), characterized by acute, painless vision loss due to inadequate blood circulation in the optic nerve head, leading to disc swelling [4]. Its incidence is 2.3 to 10.2 per 100,000 people aged 50 and older each year [4]. The pathophysiology of NAION is often described as compartment syndrome, where venous congestion, edema, and increased pressure further impair perfusion, particularly in individuals with crowded optic discs or *discs at risk*, an anatomical trait that increases susceptibility [1].

This possible association with GLP-1 RAs may be related to their strong glycemic-lowering effect. Rapid improvements in glycemic control have been linked to early worsening of diabetic retinopathy and, in some cases, NAION [1].

This suggests that GLP-1 RA-related NAION might be a consequence of metabolic shifts rather than a direct toxic effect [1]. This mechanism is supported by reports of transient worsening of diabetic retinopathy and NAION following bariatric surgery or intensive diabetes treatment [1]. Initial reports, including a single case of bilateral NAION following semaglutide use and findings from a neuro ophthalmology clinic cohort, prompted further investigation [1]. While some large population-based studies found no significant imbalances, others observed a two- to threefold higher risk of NAION with semaglutide [1]. Given the increasing use and superior efficacy of semaglutide compared to older alternatives, understanding this potential link is important for patient safety and clinical decision-making [1,4].

Therefore, the objective of this review is to systematically synthesize and examine the available evidence regarding the association between semaglutide, and more broadly, GLP-1 RAs, and NAION.

## Review

Methodology

Research Question (PICO Framework)

This review focuses on individuals receiving treatment with GLP-1 RAs, such as semaglutide, primarily for the management of type 2 diabetes or weight loss. The intervention of interest is exposure to GLP-1 RAs, while the comparator group includes individuals without GLP-1 RA exposure or those treated with other non-GLP-1 antidiabetic agents, when applicable. The primary outcome evaluated is the development of NAION.

Final Research Question

Is there an association between the use of GLP-1 RAs such as semaglutide and the development of NAION in adults?

Inclusion Criteria

Studies were eligible for inclusion if they involved patients exposed to GLP-1 RAs such as semaglutide. Eligible studies were required to report cases of NAION or other forms of ION occurring after GLP-1 agonist use. We considered various study designs, including cohort studies, case series, case-control studies, retrospective analyses, and randomized controlled trials (RCTs). To ensure relevance and recency, only studies published in English between 2015 and 2025 were included. Additionally, studies needed to provide sufficient detail regarding the temporal relationship between drug exposure and the diagnosis of NAION.

Exclusion Criteria

We excluded studies involving non-human subjects, including animal or in vitro experiments. Narrative reviews, editorials, letters, and commentaries without original clinical data were also excluded. Studies focusing on arteritic, traumatic, or other non-ischemic forms of optic neuropathy were not considered. Furthermore, reports without clear documentation of GLP-1 RA exposure or those published in languages other than English were excluded. Finally, studies lacking adequate clinical detail to assess the relationship between exposure and outcome were omitted from this review.

Information Sources

The literature search was conducted in accordance with Preferred Reporting Items for Systematic Reviews and Meta-Analyses (PRISMA) reporting guidelines across four major electronic databases: PubMed, ScienceDirect, Google Scholar, and the Cochrane Library on June 26, 2025. 

Search Strategy

A comprehensive literature search was performed across multiple databases using predefined keywords and Boolean operators. In PubMed, the search string used was: (semaglutide [Title/Abstract]) OR (ozempic [Title/Abstract]) AND (non-arteritic anterior ischemic optic neuropathy [Title/Abstract]) OR (ischemic optic neuropathy [Title/Abstract]). Filters applied included publication year within the past 10 years, free full-text availability, and article types such as meta-analyses, clinical trials, observational studies, and RCTs. Results were sorted by best match.

For Google Scholar, the search was conducted using the string: semaglutide OR ozempic AND non-arteritic anterior ischemic optic neuropathy OR ischemic optic neuropathy. Filters included publications from 2014 to 2025 and English language.

Similarly, the Cochrane Library was searched using the terms: semaglutide OR ozempic AND non-arteritic anterior ischemic optic neuropathy OR ischemic optic neuropathy. Filters applied were limited to publications from 2015 to 2025 in English, with sources including PubMed and Embase. Records that were not fully accessible were manually excluded from the screening process.

Study Selection Process

**Figure 1:**
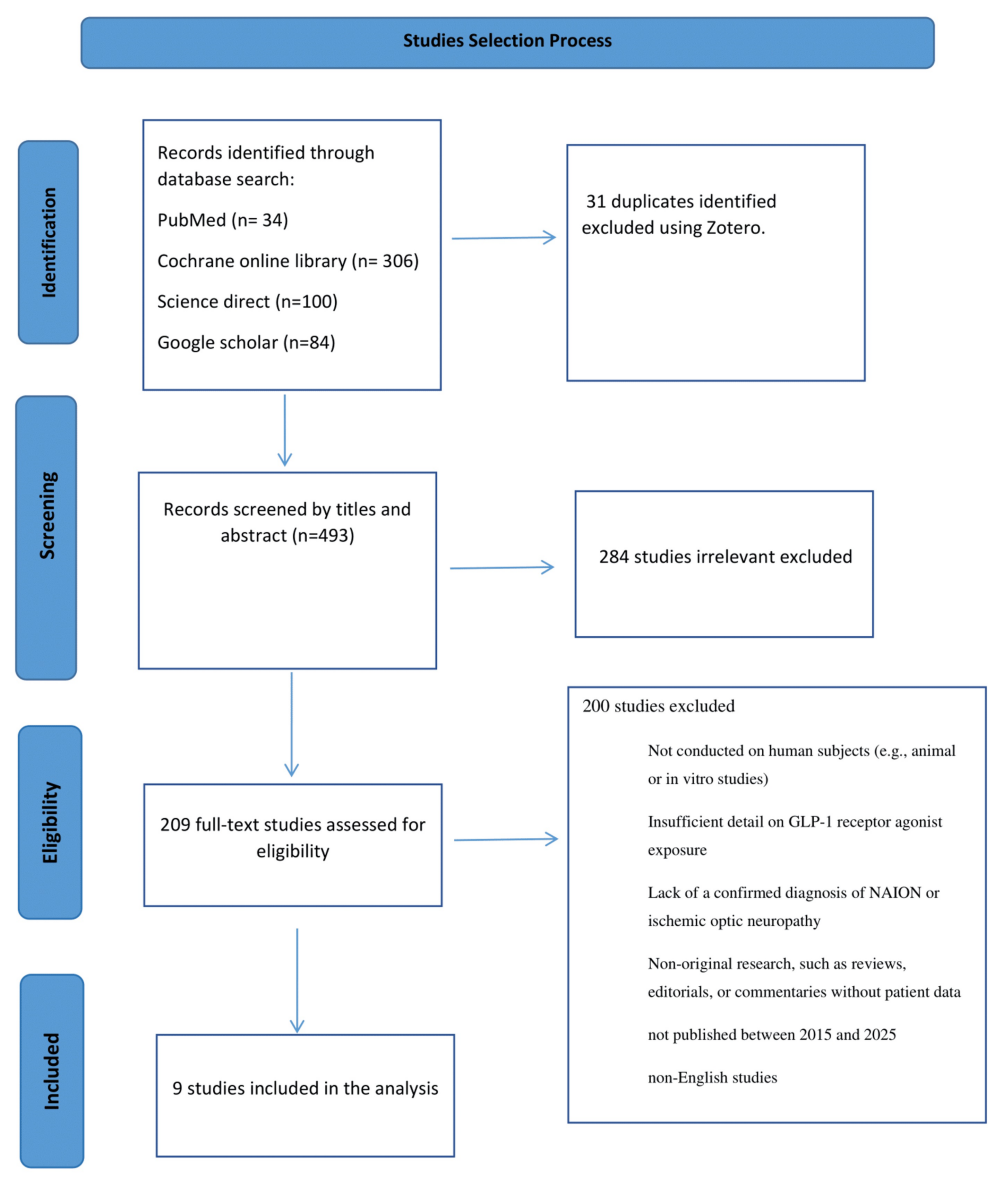
PRISMA flow diagram. PRISMA, Preferred Reporting Items for Systematic Reviews and Meta-Analyses

Two different reviewers performed the study selection process, as outlined in the PRISMA reporting guidelines (Figure 1). Afterward, a third researcher helped to finish making the selection of the articles involved in this review. 

Data Extraction

Data extraction was conducted independently by two reviewers using a standardized and pre-piloted data extraction form designed specifically for this review. For each included study, the following data items were collected: author(s), year of publication, country of study, study design, patient demographics (e.g., age, sex), indication for GLP-1 RA use (e.g., T2DM, obesity), specific GLP-1 agonist administered (e.g., semaglutide), and key outcomes (Table 1).

**Table 1:** Data extracted. NAION, non-arteritic anterior ischemic optic neuropathy; BMI, body mass index; GLP-1 RAs, glucagon like peptide-1 receptor agonist

Study ID	Country of study	Study design	Patient age	Indications for GLP-1 RA use	Specific GLP-1 agonist administered	Key outcome
Abbass et al., 2025 [5]	United States	Retrospective matched cohort study	Patients ≥12 years old	Type 2 diabetes mellitus (T2DM) and overweight or obese (high BMI)	Semaglutide (primary analysis). Any GLP-1 RA (secondary analysis).	No significant increase in risk of NAION or ischemic optic neuropathy (ION) in patients taking semaglutide or GLP-1 RAs compared to T2DM or high BMI controls.
Ahmadi and Hamann, 2025 [6]	Denmark	Retrospective case series (single centre)	Over 47 years	Obesity (Cases 1, 2, and 4)	Semaglutide (Wegovy/Ozempic)	The observations suggest that individuals with small Bruch’s membrane opening (BMO) diameter may be at risk of developing NAION during semaglutide treatment.
Cai et al., 2025 [7]	United States (OHDSI network, 14 databases)	Retrospective study	Median age 50-69 years	Type 2 diabetes (T2D)	Semaglutide (target exposure)	The study suggests a modest increase in the risk of NAION among individuals with T2D associated with semaglutide use, which is smaller than previously reported.
Chou et al., 2025 [4]	Multinational	Retrospective cohort study	Mean age 48–63 years	T2DM	Semaglutide	Avoidance of semaglutide based solely on concerns regarding the risk of NAION may not be warranted, as its potential benefits for blood glucose control and cardiovascular health likely outweigh these potential risks.
Grauslund et al., 2024 [8]	Denmark	Five-year longitudinal cohort study	Median age: 65 years	T2D	Semaglutide (Ozempic)	During five years of observation of all persons with T2D in Denmark, use of once-weekly semaglutide independently more than doubled the risk of NAION (hazard ratio (HR) of 2.19, 95% confidence interval (CI) 1.54-3.12; *P* < 0.001).
Klonoff et al., 2024 [9]	United States	Seven different retrospective real-world cohort analyses	18 and over	T2DM	Semaglutide	The study provides robust real-world evidence that GLP-1 RAs (including semaglutide) do not increase NAION risk when confounding factors are accounted for.
Poonja and Chen, 2025 [10]	United States	Retrospective matched cohort study	Adults (≥18 years)	T2DM, Obesity	Semaglutide (primary focus)	Significantly higher HR of NAION in patients prescribed semaglutide for both T2DM and obesity.
Simonsen et al., 2025 [11]	Denmark and Norway	Cohort study	Median age of the cohorts was 65 years	T2D and obesity	Semaglutide	The study observed a statistically significant increased risk of NAION among semaglutide users for T2D.
Zhao et al., 2025 [12]	Multinational	Retrospective pharmacovigilance study	Over 18 years	T2D and obesity	Semaglutide	There is a significant difference in ocular adverse drug events risk between subcutaneous and oral semaglutide, which provides evidence for dosage form selection and risk monitoring in clinical use.

Quality Assessment

The included studies did not include any RCTs; hence, the quality was assessed using Risk of Bias in Non-randomized Studies (ROBINS-I; Table 2).

**Table 2:** Quality assessment using the ROBINS-I tool. ROBINS-I, Risk of Bias in Non-randomized Studies-I

Study	Confounding	Selection of participants	Classification of interventions	Deviations from intended interventions	Missing data	Measurement of outcomes	Reported results
Abbass et al., 2025 [5]	Low risk	Low risk	Moderate risk	Low risk	Low risk	Moderate risk	Low risk
Ahmadi and Hamann, 2025 [6]	Low risk	Moderate	Low risk	Low risk	Low risk	Moderate risk	Low risk
Cai et al., 2025 [7]	Low risk	Low risk	Low risk	Low risk	Low risk	Moderate risk	Low risk
Chou et al., 2025 [4]	Low risk	Low risk	Low risk	Low risk	Low risk	Moderate risk	Low risk
Grauslund et al., 2024 [8]	Moderate risk	Low risk	Low risk	Low risk	Low risk	Moderate risk	Low risk
Klonoff et al., 2024 [9]	Low risk	Low risk	Low risk	Moderate risk	Low risk	Moderate risk	Low risk
Poonja and Chen, 2025 [10]	High risk	Low risk	Low risk	Moderate risk	Low risk	Moderate risk	Low risk
Simonsen et al., 2025 [11]	Moderate risk	Low risk	Low risk	Moderate risk	Low risk	Moderate risk	Low risk
Zhao et al., 2025 [12]	Moderate risk	Moderate risk	Low risk	Low risk	High risk	Moderate risk	Low risk

Data Synthesis

Data from the included studies were synthesized narratively due to substantial heterogeneity in study design, outcome reporting, and population characteristics. The literature comprised a mixture of case reports, retrospective cohort studies, large real-world database analyses, and meta-analyses, with varying definitions and diagnostic criteria for NAION. Additionally, the available studies differed in their exposure measurement (e.g., semaglutide dose), comparator groups, and risk adjustment methods. Because of these methodological differences and the predominance of qualitative and observational data, a quantitative meta-analysis was not feasible. Instead, findings were summarized descriptively, highlighting patterns of association, reported risk estimates, and clinical interpretations across study types.

Results

Study Selection

The study selection process began with identifying records from various databases, resulting in 524 records. After excluding 31 duplicates and 284 irrelevant studies, 209 full-text studies were assessed for eligibility. Ultimately, 200 studies were excluded due to various criteria, including non-human subjects and insufficient data. Finally, nine studies were included in the analysis, focusing on GLP-1 RA exposure and related conditions.

Quality Assessment

The quality of the included studies was assessed using the ROBINS-I tool, revealing varying levels of risk across different domains. Most studies showed low risk for confounding and selection of participants. However, several studies exhibited moderate risk in the classification of interventions, deviations from intended interventions, and measurement of outcomes. This could have affected the incidence of how developing NAION was related to the use semaglutide. Notably, Zhao et al. [12] demonstrated high risk for missing data, potentially impacting the reliability of their findings. Despite these variations, all studies were deemed low risk for reported results, suggesting overall robustness in how outcomes were communicated.

Synthesis of Findings

The association between the use of GLP-1 RAs, such as semaglutide (including Ozempic), and the development of NAION in adults presents conflicting evidence across large-scale studies. Some extensive analyses suggest an increased risk. A Danish cohort study, which included over 424,000 people with type 2 diabetes, found that semaglutide use independently more than doubled the risk of NAION (hazard ratio (HR) = 2.19, 95% confidence interval (CI) 1.54-3.12; *P* < 0.001) [8]. This result was mirrored in a cohort study comparing semaglutide with sodium-glucose cotransporter 2 (SGLT-2) inhibitors, conducted among Danish and Norwegian participants. The study reported a pooled, adjusted HR of 2.81 (95% CI: 1.67-4.75) for the risk of NAION development [11]. In contrast, a large retrospective analysis of 14 databases identified a small but statistically significant increase in the incidence rate of NAION associated with semaglutide exposure. This was based on a self-controlled case-series analysis, which reported a meta-analytic incidence rate ratio (IRR) ranging from 1.32 to 1.50 (*P* < 0.001) [7]. There was also increased risk of semaglutide with cohort analysis (HR 2.27, 95% CI 1.16-4.46) in this study as compared to empagliflozin [7]. Moreover, an analysis of data collected by the Food and Drug Administration (FDA) Adverse Event Reporting System (FAERS) reported the signs of ION with subcutaneous semaglutide (Reporting Odds Ratio (ROR) 9.15) [12].

However, other significant studies indicate no statistically significant association. One retrospective matched cohort study found no significant increase in risk of NAION or ION in patients taking semaglutide or any GLP-1 RA compared to matched controls with type 2 diabetes or high BMI [5]. A multinational population-based study similarly concluded that semaglutide may not be associated with an increased risk of NAION in the general population, reporting non-significant hazard ratios across various patient subgroups and follow-up periods [4]. Moreover, a real-world cohort analysis with multiple sensitivity analyses found no significant increase in NAION risk from semaglutide or any GLP-1 RA after controlling for confounders [9].

While case reports describe the occurrence of NAION in patients on semaglutide, often in those with anatomical predispositions like a disc at risk (e.g., small Bruch's membrane opening (BMO) and crowded optic disc) [6], the precise underlying mechanism for any potential association remains unclear [7,10]. Despite these varied results, a recurring theme in the sources is that even if an increased risk exists, its absolute magnitude is low, and the well-documented benefits of GLP-1 RAs for managing diabetes and cardiovascular health likely outweigh this potential concern for most patients [7,10-12]. Further research incorporating detailed ophthalmic risk factors and examining dose-dependent effects is warranted to better understand any causal relationship [7].

Discussion

The current systematic review summarised the evidence of nine studies that examined the possible relationship between semaglutide, a GLP-1 RA, and the emergence of NAION (NAION). The results provided mixed associations as some reports confirmed statistically significant increase in risks, and others did not confirm such association. To give an example, Grauslund et al. [8] found that semaglutide (once-weekly) alone increased the risk of five-year NAION almost twice as high compared to the risk of individuals with type 2 diabetes with no treatment or intervention. Equally, a pooled adjusted hazard ratio of 2.81 was found by Simonsen et al. [11] in Danish-Norwegian cohort and by Zhao et al. [12], a strong signal of pharmacovigilance (ROR 9.15) on the subcutaneous semaglutide. Conversely, matched cohort and real-world studies conducted by Abbass et al. [5] and Klonoff et al. [9] did not show a significant relation after adjusting to confounding factors.

The mechanism through which GLP-1 RAs can contribute to NAION has not been clarified. However, it is believed that GLP-1 RAs may generate vascular alterations that affect the perfusion of the optic nerve in patients that have predisposition to this pathology. At the same time, it has been stated that strict control of glucose levels is linked with early exacerbations of retinophaty and NAION [6]. 

The heterogeneity in study design, patient demographics, exposure measurement, and outcome definitions limited the comparability of findings. Notably, Cai et al. [7] found only a modest risk increase (IRR 1.32 - 1.50), suggesting that any potential association may be smaller than initially suspected. Furthermore, some case series, such as Ahmadi & Hamann [6], indicated that anatomical predispositions like a small BMO could heighten susceptibility to NAION during semaglutide therapy. The lack of RCTs, inconsistency in diagnostic criteria, and reliance on retrospective databases further weaken causal inference. Due to these limitations, meta-analysis was not feasible.

The quality assessment presented in Table 2 demonstrated variability in methodological rigor. Retrospective cohort studies generally met most criteria, though often lacked control for confounders like vascular risk or glycemic fluctuations. Case reports and pharmacovigilance analyses were more limited, frequently missing standardized diagnostic criteria or adequate follow up. No study scored high across all domains, with several showing moderate to high risk of bias. These findings highlight the need for well designed prospective studies to better evaluate the potential association.

Despite these concerns, the absolute risk appears low, and the therapeutic benefits of semaglutide particularly its cardiovascular and metabolic advantages remain compelling [4,10]. However, clinicians should take precautions when prescribing this medication, especially in patients with predisposing ocular anatomy or vascular comorbidities. Prospective studies incorporating ophthalmic assessments are essential to establish causality and guide clinical decision-making.

## Conclusions

This systematic review evaluated the existing evidence on the potential association between semaglutide, a GLP-1 RA, and the development of NAION. The findings across nine studies were mixed, with some large-scale cohort and pharmacovigilance studies indicating a modestly increased risk, while others found no statistically significant association after controlling for confounding factors. The heterogeneity in study design, population characteristics, outcome definitions, and diagnostic accuracy limits the ability to draw definitive conclusions. While a small potential risk of NAION may exist, the absolute risk remains low, and current evidence does not support widespread changes to clinical practice. Given semaglutide’s proven efficacy in managing type 2 diabetes and obesity, its benefits likely outweigh the potential ocular risks for most patients. However, clinicians should exercise caution when prescribing semaglutide to individuals with a history or predisposing conditions that could lead to optic nerve anatomy or other vascular risk factors for NAION. Further prospective, ophthalmology-focused research is warranted to clarify causality and inform safer clinical use.
